# Promising degrees of stakeholder interaction in research for sustainable development

**DOI:** 10.1007/s11625-017-0507-4

**Published:** 2017-11-07

**Authors:** Flurina Schneider, Tobias Buser

**Affiliations:** 1 Centre for Development and Environment (CDE), University of Bern, Hallerstr. 10, 3012 Bern, Switzerland; 2 Network for Transdisciplinary Research (td-net), Swiss Academies of Arts and Sciences, Bern, Switzerland

**Keywords:** Stakeholder collaborations, Transdisciplinary research, Co-production of knowledge, Evaluation of stakeholder interaction designs

## Abstract

Stakeholder interactions are increasingly viewed as an important element of research for sustainable development. But to what extent, how, and for which goals should stakeholders be involved? In this article, we explore what degrees of stakeholder interaction show the most promise in research for sustainable development. For this purpose, we examine 16 research projects from the transdisciplinary research programme NRP 61 on sustainable water management in Switzerland. The results suggest that various degrees of stakeholder interaction can be beneficial depending on each project’s intended contribution to sustainability, the form of knowledge desired, how contested the issues are, the level of actor diversity, actors’ interests, and existing collaborations between actors. We argue that systematic reflection about these six criteria can enable tailoring stakeholder interaction processes according specific project goals and context conditions.

## Introduction

Universities are increasingly called upon to produce knowledge that is relevant for society in general and for sustainable development in particular (WCED 1987; ISSC 2012; Earth 2014; Open Working Group of the General Assembly 2015). There are also a growing number of researchers who want to contribute to sustainability transformations through their research (CASS and ProClim 1997; Tàbara and Chabay 2013; Miller et al. 2014). It is virtually uncontested that societally relevant research requires some sort of stakeholder interaction (Lang et al. 2012; van der Hel 2016). But to what extent, how, and for which goals should different stakeholders be involved in sustainability research projects? And what are promising designs for stakeholder–researcher collaborations?

Researchers, funding bodies, and stakeholders answer these questions in different ways (Mielke et al. 2016; van der Hel 2016; Wiek and Lang 2016). At one end of the spectrum, we find those who argue that stakeholder interaction is fundamental to science for sustainable development. Relevant authors refer to concepts such as Mode 2 (Nowotny et al. 2001), post-normal science (Funtowicz and Ravetz 1993), co-production of knowledge (Jasanoff 2004), transdisciplinary research (Hirsch Hadorn et al. 2006), action research (Reason and Bradbury 2001; Bradbury 2015), and participatory or collaborative research. These authors assume that investigation of real-world sustainability challenges and identification of solutions require novel ways of knowledge production, which acknowledge the complexity, uncertainty, and contested nature of sustainability challenges. At the other end of the spectrum, researchers stress the need to conduct independent, “excellent” academic research about sustainability issues. When they describe how to use their results to contribute to sustainability, they often refer to concepts such as knowledge transfer, innovation diffusion, or science communication. They assume that scientists produce new knowledge and then simply transfer it to practitioners or decision makers via the media, boundary organizations, or advisory services.

While there is considerable scientific literature arguing for one mode or the other (Hirsch Hadorn et al. 2006; Schneidewind 2009; Kueffer et al. 2012; Strohschneider 2014), there are very few studies that systematically outline and compare different ways of contributing to sustainability (Mielke et al. 2016; van der Hel 2016). This is especially the case regarding provision of practical guidelines for researchers looking to identify promising degrees of stakeholder interaction. Addressing this gap is the overall goal of this article.

Identifying promising degrees of stakeholder interaction in specific projects is important for three interrelated reasons: it enables the projects (a) to reach the intended sustainability contribution goal, (b) to deal responsibly with stakeholders' and researchers' time and resources, as high degrees of stakeholder interaction take considerable time, resources and skills, and consequently (c) to reduce the risk of participation fatigue, project failure, and friction among collaborators.

### Transdisciplinary research

One sustainability-oriented research field that *has* begun to systematize and compare different stakeholder interaction approaches is transdisciplinary research. Put simply, transdisciplinary research is a collaborative mode of knowledge production that is oriented towards specific societal challenges and integrates knowledge and perspectives from different scientific disciplines and stakeholders. Hence, stakeholder interaction processes are an important element of transdisciplinary research. While some authors view stakeholder interactions as the key criterion of transdisciplinary research, other authors emphasize different definitional elements such as the need to extend typical notions of scientific knowledge in order to account for more diverse forms of knowledge, such as normative and transformational knowledge (Grunwald 2004; Wuelser et al. 2012).

In this article, we refer to three basic concepts that have been developed in the field of transdisciplinary research:

A. Degrees of stakeholder interaction: We use this term to describe different modes of stakeholder involvement in research and what roles are attributed to them. Lower degrees of stakeholder interaction refer to cases in which stakeholders are mere recipients of knowledge (e.g. one-way information transfer). Medium degrees refer to cases in which stakeholders are consulted to express their knowledge (e.g. interview or focus group situations). Higher degrees refer to modes of collaboration in which knowledge is truly co-produced and stakeholders co-shape the research process (e.g. reciprocal learning between researchers and stakeholders, integration of different perspectives). These interaction degrees draw on Arnstein’s “ladder of participation” (Arnstein 1969), but are adapted for knowledge-production process based on Mobjörk (2010) and Stauffacher et al. (2008).
B. Research phases: We use this term to refer to a conceptual model of an ideal–typical research process. It comprises three phases: Phase A “framing the problem and research goal” (defining what are the most relevant sustainability problems and what research should/can contribute); Phase B “(co-)producing new knowledge” (conducting inter-, trans-, or disciplinary research); and Phase C “bringing results to fruition” (re-integrating the new knowledge into scientific and societal practice) (Bergmann et al. 2005; Pohl and Hirsch Hadorn 2007; Jahn et al. 2012; Lang et al. 2012). Accordingly, in each of these phases—which rotate iteratively and cyclically—stakeholder interactions serve different goals.
C. Three forms of knowledge: To account for the different forms of knowledge needed when wanting to contribute to sustainable development through research, we refer to the concepts of systems, target and transformation knowledge introduced by Swiss researchers in a manifest for Research on Sustainability and Global Change (Proclim/CASS 1997). *Systems knowledge* is analytical, descriptive or explanatory knowledge about specific sustainability problems. *Target knowledge* is normative knowledge about values and norms related to more desirable futures. *Transformation knowledge* is practical knowledge about how to transform an existing, problematic situation into a better one (Hirsch Hadorn et al. 2008; Pohl 2011; Wuelser et al. 2012; Schneider 2016). These three forms of knowledge relate to different topics and ways of knowing, but they are also very interdependent and build on each other. For example, transformation knowledge for sustainable development is based upon sound understanding of the underlying systems and value-explicit target knowledge.

### Methods, context conditions, and stakeholder interaction processes

Several researchers have investigated stakeholder interaction processes in recent years. They studied the kinds of methods and knowledge-production processes applied, the roles attributed to stakeholders and researchers, the observable outcomes, and people’s experiences (Bergmann and Schramm 2008; Stauffacher et al. 2008; Wiesmann et al. 2011; Lang et al. 2012; Renner et al. 2013; Schneider and Rist 2013; Defila and Di Giulio 2016; Siew et al. 2016). While a lot of emphasis has been placed on studying what might be promising methods and approaches, another key insight of these studies is that stakeholder interaction processes must be carefully tailored to the specific context conditions of research projects. Context conditions of distinct regions and countries can be quite different, for example, with respect to the dominant political culture (e.g. whether actors are used to freely speaking out and engaging in open dialogue with hierarchically “superior” actors), education levels (e.g. if many actors are illiterate), and social situations (e.g. conditions reflecting attitudes about the appropriate roles of different actor groups) (Wiesmann et al. 2011; Siew et al. 2016). Consequently, based on an investigation of the degrees of stakeholder interaction vis-à-vis project progress in landscape planning in Switzerland, Stauffacher et al. (2008) suggest that tailored techniques be selected and integrated to provide the foundation for inclusive interactions depending on the issue, type, goals, and phase of the decision process in question.

Lang et al. (2012) suggest that more emphasis must be placed on better understanding context conditions across various cases in order to further strengthen the quality of stakeholder interaction processes in sustainability research. However, there are very few meta-level analyses that systematically compare different approaches to stakeholder interaction in scientific research against the backdrop of different goals and context conditions (Lang et al. 2012; Brandt et al. 2013). As a consequence, there is very little published guidance regarding appropriate degrees of stakeholder interaction in different situations.

### Researchers’ rationales, epistemologies, and stakeholder interaction processes

While tailoring stakeholder interaction processes to specific project goals and context conditions is clearly vital, studies also show that researchers’ rationales and epistemologies make a difference. Mielke et al. (2016) introduced a typology of stakeholder interactions in research by distinguishing four ideal types: the technocratic, the functionalist, the neoliberal-rational, and the democratic. They differ regarding scientists’ role/identity, the objectives of stakeholder involvement, the kind of knowledge to be produced, and underlying epistemological assumptions. van der Hel (2016) developed a similar typology, but emphasized the rationales for stakeholder interaction and their consequences for practices of stakeholder involvement. She identified three overall rationales applied by different researchers: accountability (living up to societal needs and values), impact (implementation of research), and humility (acknowledgement that there are many legitimate knowledge holders other than scientists). Depending on which rationale a research project favours, different degrees and modes of stakeholder interaction appear suitable. Researchers applying the accountability rationale tend to highlight the need to involve stakeholders at the very start of a research project, so as to jointly frame the relevant sustainability problems, the kinds of knowledge capable of addressing them, and consequently, what research questions should be investigated. Researchers driven by the impact rationale tend to stress the importance of including stakeholders throughout the entire research process, so as to enable trust, ownership, and implementable knowledge. Researchers favouring the humility rationale argue for acknowledging that science is only one legitimate knowledge form among many, and there are other relevant ways of learning and understanding in the search for solutions to complex, uncertain, and contested sustainability problems. Consequently, researchers in the latter group view stakeholders as epistemic partners in the knowledge production process.

All the authors stress that these typologies are ideal types, which intermingle in practice. Nevertheless, the work of Mielke et al. (2016) and van der Hel (2016) clearly demonstrates that promising degrees and modes of stakeholder interaction cannot be defined independent of the rationales, epistemologies, and change theories of the researchers involved. These comprise researchers’ varying conceptions of what we can know; what science can contribute; what goals, approaches and methods of knowledge production are legitimate; and how knowledge and action relate to each other (Miller et al. 2008).

Against this background, the goal of our paper was to explore the stakeholder interaction processes of different research projects, so as to better understand what designs bear the most promise in specific situations. This knowledge should support researchers in devising promising transdisciplinary research designs, and support funders in evaluating them. To achieve this overall goal, we investigate the following three research questions:

1. To what degree do research projects interact with stakeholders?
2. What criteria provide indications of promising stakeholder interaction designs?
3. What degrees of stakeholder interaction are most promising in specific situations?

## Research design and method

To better understand what stakeholder interaction designs might bear the most promise under specific context conditions, we analysed stakeholder processes of research projects belonging to the Swiss National Research Programme 61 on “Sustainable water management” (NRP 61).

### The Swiss National Research Programme 61 on “Sustainable water management”

NRP 61 is one of several National Research Programmes (NRPs) funded by the Swiss National Science Foundation (SNSF). The funding scheme aims at implementing coordinated research projects that “contribute to the solution of contemporary problems of national importance” (SNF 2013). According to the SNSF website, NRPs are solution-oriented and practically relevant, interdisciplinary and transdisciplinary, and place great value on knowledge transfer and communication of results.

Within the framework of NRP 61, a total of 16 independent research projects were launched bearing the shared overall goal of developing scientific foundations and methods for sustainable management of water resources. More specifically, the research had three aims: (1) to investigate the effects of climate and social changes on water resources; (2) to examine risk, user conflict, and ecological change management from a comprehensive perspective; and (3) to develop efficient and sustainable water resource management systems (SNF 2013). Effective stakeholder interaction processes were an important element of the programme: project leaders were asked to present stakeholder interaction concepts as part of their research proposals. The quality of these concepts and their implementation were assessed in the review and annual reporting system.

The projects were funded with an overall budget of 12 million Swiss Francs for a period of 4 years, lasting from 2010 to 2013. The 16 funded projects mainly included researchers from universities and federal research institutes, but also specialists from a private research institute, an NGO, and private consultancy offices. People from a broad range of disciplines were engaged in the projects, including experts in the natural and social sciences, engineering, and, to a lesser extent, humanities and economics.

Table 1 provides an overview of the 16 research projects, their topics, and their intended sustainability contributions. More information on the projects can be found on the programme website (http://www.nfp61.ch/en).

**Table 1:** Overview of the 16 transdisciplinary research projects

Project name	Research topic	Sustainability contribution goal
NELAK	Lakes as a consequence of melting glaciers: opportunities and risks	Providing a knowledge base on lakes resulting from melting glaciers, so as to facilitate early, integrated, and participatory planning
AGWAM	Increasing water scarcity for Swiss agriculture	Developing recommendations for addressing water scarcity under different climate, price, and policy scenarios, and identifying suitable strategies for maintaining profitability without compromising environmental standards
GW-TREND	Groundwater shortage due to climate change?	Better understanding the sensitivity of aquifers to climate change
FUGE	Glacier retreat—still sufficient water for hydroelectric power production?	Providing knowledge about whether and how glacier retreat will affect hydroelectric power production
MONTANAQUA	Water management in times of scarcity and climate change	Developing sustainable water governance strategies together with all relevant stakeholders
WATERCHANNELS	Water channels: a model for sustainable water use	Promoting traditional water channels by pointing out their ecological and socio-cultural benefits and identifying under what circumstances it makes sense to retain or reactivate water channels and underlying meadow-irrigation systems
GW-TEMP	Understanding how climate change is affecting groundwater	Better understanding the possible effects of climate change on groundwater so as to prevent negative impacts on water infrastructure
SEDRIVER	More floods—more sediment transport—fewer fish?	Better understanding possible effects of climate change on the transport of sediment and on river trout in mountain streams. The improved model should assist experts and decision-makers in assessing risks in Alpine catchment areas
SWIP	Sustainable water infrastructure planning	Developing an improved water infrastructure planning procedure that balances economic, ecological, and social aspects
IWAGO	Towards integrative water governance	Developing strategies and tools for a more holistic and collaborative approach to water management in Switzerland
DROUGHT-CH	Are we prepared for droughts?	Developing a platform for improving early warning of drought periods and their consequences
SWISSKARST	Karstic waters: a water resource for the future?	Providing a national inventory of karst aquifers as a knowledge base for more sustainable water management in karstic regions
IWAQA	Integrated river water quality management	Developing a prototype for decision-making procedures in integrated river management
RIBACLIM	Is drinking water derived from rivers still clean enough?	Better understanding the possible risks of climate change impacts on riverbank filtration to identify whether action will be needed to maintain drinking water quality
SACFLOOD	How are flood hazards in the Alps evolving?	Better understanding the relationship between precipitation, the storage capacity of soils, and conditions underground so as to improve the reliability of flood estimates
HYDROSERV	Sustainable safeguarding of water resources	Better understanding the whole value chain of hydrological ecosystem services and developing decision-making tools for policymakers politicians

### Research procedure

The research consisted of five steps. First, we empirically investigated how the 16 research projects within NRP 61 designed, implemented, and perceived collaboration with stakeholders. To do so, we conducted semi-structured interviews with members from every project. In most cases, we interviewed the primary investigator (14 interviews). In some cases, we also interviewed the person responsible for stakeholder engagement (3 interviews). In the interviews, timelines of the projects’ stakeholder interaction processes were drawn. The timelines were structured according to the three project phases: problem and goal definition, production of new knowledge, and bringing results to fruition. When jointly drawing the timelines, we identified what stakeholders were involved in which activities, what roles the researchers and stakeholders had, what methods were applied, how the stakeholders influenced the research process, and what outcomes were achieved. We also asked the interviewees about the extent to which they achieved their sustainability impact goals, whether they were satisfied with the chosen stakeholder interaction designs, what challenges they encountered, and what interaction approaches might have been more fruitful. Hence, we focused more on what the projects accomplished, rather than on their initial plans and proposals. The interviews were audio recorded, transcribed, and analysed according to the rules of qualitative content analysis (Flick 2005). In addition, we assessed the project descriptions, project videos, professional articles, and progress reports. To deepen our insights, we also conducted a total of two workshops with four particular projects that featured a high degree of stakeholder interaction—the outcome of one of these workshops has been published by Renner et al. (2013).

Second, to structure the variety of different approaches and methods, we assessed the degree of stakeholder interaction of each project according to a scale adapted from Stauffacher et al. (2008). In so doing, we took into account Mobjörk’s work on consultative and co-productive ways of transdisciplinarity (Mobjörk, 2010) and Pohl’s insights on respecting and integrating different perspectives (Pohl, 2011). These comprise the following: (1) informing, (2) informing with feedback possibility, (3) consultation (few perspectives considered), (4) consultation (broad spectrum of perspectives considered), (5) co-production (some elements co-produced), (6) co-production (main elements co-produced). This assessment was done for each of the three main phases of a research project: goal and problem definition, production of new knowledge, and bringing results to fruition. In the process, we did not classify single methods, but rather the overall interaction approach; thus a project classified as co-production generally also involves informing and consultation elements. The criteria for assigning particular degrees of interaction to individual projects can be seen below in Table 2.

**Table 2:** Overview of the criteria used to assign different degrees of stakeholder interaction over the three phases of transdisciplinary research

Interaction degree		Problem-framing and goal-definition phase	Knowledge-production phase	Bringing-new-knowledge-to-fruition phase
Co-production	6	Problem and goal co-framed by scientists and stakeholders; main elements of the proposal are co-designed	Co-production of knowledge including deliberation and integration of all relevant stakeholder perspectives regarding main project elements	Co-producing main project outcomes and jointly constructing follow-up structures/actions, and engaging in societal learning processes
	5	Problem and (overall) goal co-framed by scientists and stakeholders; some elements of the proposal are co-designed	Co-production of knowledge including deliberation and integration of all relevant stakeholder perspectives regarding some project elements	Co-producing some project outcomes and/or jointly constructing follow-up structures/actions, and/or engaging in societal-learning processes
Consultation	4	Problem and goal framed by scientists; broad consultation of stakeholders leading to minor thematic adjustments of the proposal dealing with different stakeholders’ perspectives and priorities	Knowledge production by scientists, taking into account various stakeholders’ knowledge and perspectives. A wide range of stakeholders are consulted, but the knowledge is structured according to the scientists’ concepts	A wide range of stakeholders is consulted to discuss research results. The stakeholders’ perspectives influence final interpretations and recommendations
	3	Problem and goal framed by scientists; consultation of some stakeholders leading to minor thematic adjustments of the proposal	Knowledge production by scientists; some key stakeholders are informed and consulted for fine-tuning	Stakeholders are informed and final results and recommendations are jointly discussed
Informing	2	Problem and goal framed by scientists; a few stakeholders are informed about the project and feedback is encouraged. Stakeholder interactions influence logistical issues, but not project goals	Knowledge production by scientists; some stakeholders are informed and given an opportunity to provide feedback, e.g. in individual meetings, but they have hardly any influence on knowledge production	Stakeholders are informed about final results by means of articles and at meetings that offer a chance to clarify questions
	1	Problem and goal framed by scientists; a few stakeholders are informed about the project. Stakeholder interactions do not influence the proposal	Knowledge production by scientists; some stakeholders are informed about the status of the project	Stakeholders are informed about final results by means of articles in professional journals or newspapers

Third, in an effort to better understand what degrees of stakeholder interaction are most promising, we further evaluated the interviews to find out what factors the interviewees directly or indirectly associated with their perceived success or challenges. Based on this analysis, we identified six criteria that might plausibly explain why certain degrees of interaction were more or less successful. We then applied these criteria in an additional analytical round, investigating their possible manifestations across all 16 projects. The results were then summarized in multi-criteria tables.

Fourth, based on these multi-criteria tables, we conducted a qualitative pattern analysis looking for groups of projects featuring similar manifestations of the six criteria.

Fifth, we assessed whether the identified groups of projects (A–F) had similar degrees of stakeholder interaction, or whether differences could be explained through reported challenges. Based on this analysis, we identified promising degrees of stakeholder interactions through the three project phases.

This analysis was validated through a written consultation and a workshop to which all project representatives were invited.

## Results

In the following sections, we present the results of our research according to the three research questions.

### Diversities of stakeholder interaction degrees

Our analysis of the interviews and documents of the 16 projects revealed a wide variety of stakeholder interaction approaches. They ranged from rather classical research designs with limited stakeholder interactions, to complex collaborative designs in which stakeholders were part of the research team and/or co-produced knowledge together with researchers throughout all phases. Accordingly, the applied methods ranged from information tools such as letters and reports, to methods that enabled knowledge exchange and co-production of knowledge such as workshop series and field days. In some cases, stakeholder interactions had considerable impact on the research process and outcomes (e.g. leading to reframed project goals or adapted integration concepts). In others cases, no influence on the research was reported.

**Fig. 1:**
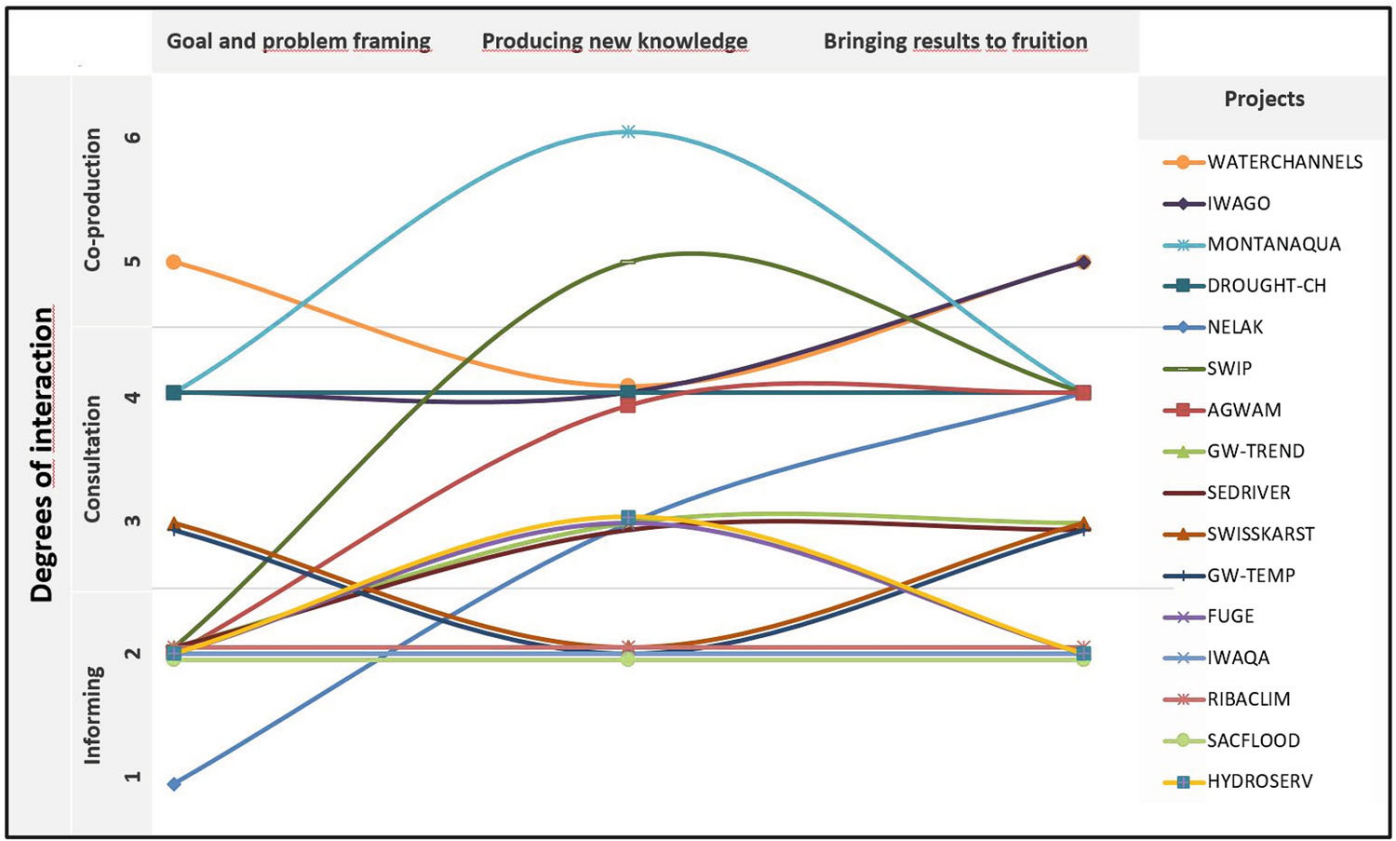
Degree of stakeholder interaction over the three research phases of the 16 projects investigated

Classifying the projects’ methods and approaches according to six different degrees of stakeholder interaction enabled a better overview of the diversity of approaches and methods (Fig. 1). Figure 1 shows that the projects differed not only regarding their average degree of stakeholder interaction, but also regarding their degree of interaction over time. Overall, we identified 11 different forms of stakeholder interaction over the three project phases. Some projects displayed consistently lower or higher degrees of interaction. Other projects displayed substantial internal variation, with higher levels of interaction at the beginning, middle, and/or end of the project.

When discussing the suitability of the stakeholder interaction approaches together with the interviewees, we found that the project representatives’ satisfaction with the chosen approach did not directly depend on the intensity of stakeholder interaction, whether its average level or development over different project phases. In other words, representatives of projects displaying very different degrees of intensity were variously satisfied or unsatisfied with their approaches. This was also the case regarding interviewees’ perception of whether projects achieved their intended sustainability impact goals or not.

### Criteria for identifying promising degrees of stakeholder interaction

Investigating how interviewees explained the perceived success or challenges of their stakeholder interaction designs, we identified the following six criteria: (1) intended sustainability contribution, (2) knowledge forms to be produced, (3) contestation, (4) actor diversity, (5) actor interest, and (6) existing collaborations.

In the following, we detail these six criteria and discuss the manifestations we found among the 16 research projects (see Table 3 for overview).

**Table 3:**
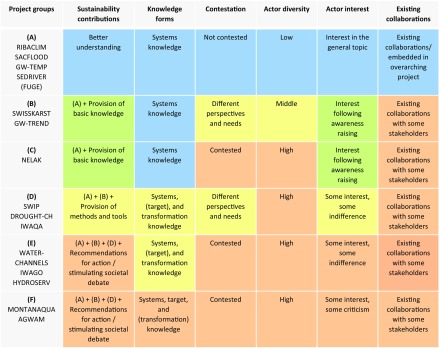
Overview of the 16 projects assessed according to six criteria for promising degrees of stakeholder interaction and grouped by similar situations (A–F)

The intended sustainability contributions are additive. Specifically, all the projects (groups A–F) sought to better understand an issue. However, only those in groups B–F additionally sought to contribute a knowledge base, while only those in group C further sought to contribute a new method or tool, etc. The colours indicate whether a lower or higher degree of stakeholder interaction is promising (from blue low, to green, yellow, and red high)

#### Criterion 1: Intended sustainability contribution

The first key criterion relates to the question of how a research project seeks to contribute to more sustainable development. This criterion reflects the epistemic assumptions of the project, its change theories, and its impact goals.

Among the NRP 61 projects, we found four different categories of intended sustainability contributions. The four categories varied strongly regarding how the links between knowledge and action were conceptualized and perceived. They could be summarized as follows: (a) Create better understanding: five projects aimed to enable better understanding of certain problem situations, without intending to bring about action among societal stakeholders (e.g. GW-TEMP investigated the possible effects of climate change on manganese fallout in groundwater; societal action might only be needed in the event that manganese fallout increases substantially). (b) Provisioning of basic knowledge: besides enabling better understanding of an issue, three projects additionally aimed to contribute a knowledge base for interested stakeholders (e.g. SWISSKARST aimed at providing a national inventory of karst aquifers as evidence for water managers). (c) Provisioning of methods and tools: three projects aimed to contribute not only new understanding and knowledge, but also fully fledged decision-making or planning tools (e.g. SWIP sought to contribute a water infrastructure planning procedure that balances economic, ecological, and social criteria). (d) Recommendations for action/ stimulation of societal debate: five projects aimed to have a more direct impact on society either by developing specific recommendations for action, or by stimulating societal debate and reflection on a contested issue (e.g. MONTANAQUA aimed to develop strategies for more sustainable water governance taking into account various stakeholder perspectives). See Table 2 for an overview of the intended sustainability contributions of all projects.

Overall, analysis of our sample showed that degrees of stakeholder interaction increased from category (a)–(d). In other words, the lowest degrees were needed in cases where projects solely aimed to better understand an issue. Higher degrees of interaction appeared necessary in cases where projects sought to trigger action directly.

#### Criterion 2: Knowledge forms to be produced

The form of knowledge missing and thought to be needed to effectively address the investigated sustainability problem was revealed to be the second key criterion. As introduced in Sect.1.1, sustainability transformations require systems, target, and transformation knowledge. But in specific situations, the lack of one or the other type of knowledge can be more critical for fostering more sustainable development (e.g. the sustainability problem might be well understood, but there might be lack of knowledge of how to achieve a more sustainable situation). Moreover, it depended on the epistemologies of researchers and whether they perceived production of systems knowledge as the sole possible knowledge goal, or if target and transformation knowledge are also perceived as part of a scientific project.

In our sample, production of systems knowledge only was perceived as most important in half of the projects, while the other half also strove to generate target and/or transformation knowledge. For example, to prevent negative impacts on water infrastructure, GW-TEMP sought to provide systems knowledge about the effects of climate change. MONTANAQUA perceived that development of strategies for more sustainable water governance required not only better understanding of systemic relations, but also envisioning of more desirable futures (target knowledge) and identification of tools to achieve these futures (transformation knowledge).

Our analysis revealed that the production of all three forms of knowledge could require stakeholder interactions. However, higher degrees of stakeholder interaction appeared to be particularly important in the case of target and transformation knowledge, as the former addresses contested societal values and norms while the latter addresses inducing and changing societal practices. In both cases, stakeholders’ knowledge and their ability to act were crucial. As a result, co-production approaches displayed the most promise.

#### Criterion 3: Contestation

A third important criterion was whether and how the addressed sustainability problems and striven-for knowledge forms were subject to societal contestation. Contestation can accompany all three forms of knowledge: systems, target, and transformation knowledge.

Five of our investigated projects described their topics as uncontested. For example, in the case of GW-TEMP, it was essentially uncontested that manganese outfalls can damage water infrastructure and that, therefore, better understanding of the effects of climate change on manganese outfalls could help to prevent such damages. Five other projects reported that their topics were not really contested, but that different stakeholders tended to have distinct perceptions and needs regarding the issue. For example, better forecasting of soil humidity was important to various stakeholders in the DROUGHT-CH project, but farmers were interested in different parameters than tourism operators or natural hazard authorities. Six projects viewed their sustainability problems as fundamentally contested. This was particularly the case in projects addressing questions of water distribution in times of scarcity, for example in AGWAM or MONTANAQUA.

The implications of an issue’s level of contestation can only be defined when also considering the first two criteria identified, i.e. how and with what type of knowledge a given research project intends to contribute to more sustainable development. This is particularly true regarding higher levels of contestation. In such situations, some projects opted to concentrate on generating systems knowledge and producing scientific evidence without stakeholder collaboration. Other projects, particularly those seeking to generate target and transformation knowledge, organized intense stakeholder processes in order to acknowledge the heterogeneity of existing perspectives or to enable stakeholders to jointly create new visions. However, our results showed that more contested topics required more carefully designed processes of stakeholder interaction. Working in conflictive fields requires sound knowledge of actors’ power constellations, needs, and fears. It also requires that researchers have skills in moderating and mediating.

#### Criterion 4: Actor diversity

The diversity of actors involved in sustainability problems was also found to be key to identifying promising degrees of stakeholder interaction. This refers to the number and heterogeneity of stakeholders who can affect, are directly affected or are otherwise involved in a given sustainability issue. Together with the criterion of contestation, actor diversity can be seen as an actor-centred proxy for the complexity of an issue.

We grouped the 16 projects according to three levels of diversity: Projects with only a few actors from one sector were considered low actor diversity (5 projects), e.g. when only the drinking water authorities of one Swiss canton were involved. Projects with a greater number of actors from related sectors were classified as medium diversity (2 projects), e.g. when the water authorities of many Swiss cantons were involved. Projects with a broad range of actors from many different sectors were considered high diversity (9 projects), e.g. when many different water user groups were involved such as agriculture, households, tourism, administration, hydropower, and industry.

With some exceptions, higher actor diversity tended to be accompanied by higher levels of contestation. However, in all cases—even in relatively uncontested situations—higher numbers of interactions and greater heterogeneity of actors called for more intense stakeholder engagement approaches. Spontaneous, occasional, and informal contact for the purpose of information sharing and feedback appeared very promising when only a few actors were involved. However, when many different actor groups had a stake, interaction events generally required more careful planning and facilitation to enable not only information transfer, but also broader consultations, knowledge exchange, and joint development of new knowledge. In such cases, not only were the interactions between researchers and stakeholders important, so too were opportunities for different stakeholders to meet and deliberate the issues at hand.

#### Criterion 5: Actor interest

This criterion involves stakeholders’ level of interest in the sustainability contribution goal of a project and the knowledge they want to produce.

In five of the investigated projects, key stakeholders were interested in the general project topic (e.g. preservation of safe drinking water), but not necessarily in the specific, relatively technical research questions. In three other projects, stakeholders became interested in the project goals only after researchers had invested considerable energy in raising awareness (e.g. about the risks of climate change-induced glacial lake outbursts). As actor diversity increased, so too did the diversity of interests. In this way, eight projects reported that some stakeholders demonstrated interest while others were somewhat indifferent and/or even critical (e.g. regarding governance issues such as redistribution of water rights).

Actors’ interest levels strongly influenced the degree of promising stakeholder interaction. If important stakeholders were more indifferent or critical, the stakeholder interaction processes had to be designed with greater care. In general, stakeholder processes also needed to be more intense in cases of particularly indifferent/critical stakeholders. For example, written communicate of information seldom proved adequate in such cases. Instead, face-to-face meetings and other more interactive forms of knowledge exchange and learning appeared more promising.

#### Criterion 6: Existing collaborations

This criterion addresses the history of a given project and how stakeholder interactions are embedded. On the one hand, it concerns researchers’ possible long-term contacts with stakeholders. On the other, it concerns whether a given project is embedded in a broader project or programme featuring intense stakeholder interaction.

Five of the projects we investigated built on existing long-term collaborations with all stakeholders that were perceived as relevant. Eleven projects involved some stakeholders who were previously unknown to the researchers and/or who did not know each other.

In the first case, projects displayed satisfactory stakeholder processes even with rather low degrees of interaction. As the researchers knew the stakeholders from earlier projects, there was already enough mutual understanding to easily agree on research priorities and desirable sustainability contributions. Based on their prior collaboration, the stakeholders trusted the researchers to produce relevant outcomes. Notably, this situation only emerged when projects involved collaborations with a small number of stakeholders (low actor diversity). As the number of new collaborations increased, more time-consuming and intense stakeholder engagement processes were required. Particularly in situations of high contestation, this meant that extensive consultations and/or knowledge co-production events were needed even just to establish a joint understanding of the sustainability problem and to define project goals.

In summary, low-intensity stakeholder engagement processes show promise under the following conditions: the researchers only aim to improve understanding of an issue (without directly inducing actions); only systems knowledge is lacking; the sustainability problem is uncontested; few actors are involved; all the actors demonstrate interest in the new knowledge; and, most importantly, the researchers and stakeholders have collaborated previously. By contrast, high-intensity stakeholder engagement processes are needed under the following conditions: there is a lack of systems, target, and transformation knowledge; the sustainability problem is contested; many stakeholders are involved and/or some are fairly critical of the research; and little or no previous collaboration exists between the researchers and the stakeholders. At the same time, it is not merely individual criteria, but rather their combination that is particularly relevant.

### Promising degrees of stakeholder interaction in specific situations

Our pattern analysis of the 16 projects revealed six groups of projects with similar characteristics (situations A–F; see Table 3 for an overview).

*Situation A* comprises five projects (RIBACLIM, SACFLOOD, GW-TEMP, SEDRIVER, and, in part, FUGE) that sought to generate systems knowledge to better understand specific aspects of relatively uncontested environmental problems in contexts of low actor diversity. In these situations, the visions of and/or actions needed for a more sustainable future appeared relatively clear—or were considered out of scope. The projects built on existing collaborations with directly concerned stakeholders or were linked to other projects with established collaborators. These stakeholders were interested, on balance, in the general research topic.

For example, GW-TEMP aimed to better understand how climate change is affecting manganese outfalls in groundwater. Researchers identified related systems knowledge as a key research gap. The research was of interest to drinking water authorities because manganese outfalls can damage water infrastructure. The research addressed possible future threats, and water authorities were only interested in whether any negative effect could be shown, as only this would require action. The situation appeared relatively uncontested.

In such projects, consistently low degrees of stakeholder interaction across all three project phases appeared sufficient (information sharing and feedback opportunities). Some projects of this type that had formed an accompanying group reported that stakeholders showed only limited interest in more intense forms of interaction. Stakeholders mainly wanted to be informed about the project status and the results.

*Situation B* comprises two projects (SWISSKARST, GW-TREND) that aimed not only to better understand an issue, but also sought to generate aggregated systems knowledge that would enable public authorities to manage water resources more sustainably. For example, SWISSKARST sought to provide a national inventory of karst aquifers as a knowledge base for more sustainable water management in karstic regions. Both projects reported that the potential target actors (medium diversity) only grew interested in the knowledge products following awareness-raising efforts. To gain the interest of a wider range of stakeholders, it was necessary to raise awareness by presenting preliminary results and discussing their possible implications.

In such projects, relatively low degrees of stakeholder interaction appeared sufficient in the knowledge production phase. However, in contrast to Situation A projects, more intense interactions were needed at the beginning or end in order to raise awareness, to jointly frame the problem, or to bring the results to fruition.

*Situation C* only comprises one project (NELAK). The characteristics of this project were similar to those of Situation B, but with the addition of high actor diversity and contestation. The project aimed at producing aggregated, interdisciplinary systems knowledge about risks currently absent from the policy agenda: namely, the formation of lakes in the Alps as a consequence of climate change and melting glaciers. The generated knowledge base was intended to facilitate early, integrated, and participatory planning. Hence, awareness-raising efforts among concerned actors were crucial. Many different stakeholders could be affected and the implications of the generated knowledge regarding societal aims and possible measures were controversial. However, the researchers did not desire to address these controversies within the project in greater depth.

In this project, a steadily rising degree of stakeholder interaction appeared promising. Since the stakeholders were unaware of the possible risks, the researchers initially framed their project goals without stakeholder interaction. However, once the knowledge production got going, the researchers began to interact with a few highly affected actors in personal face-to-face meetings, so as to raise their awareness of the issues. Towards the end of the project, they organized consultative workshops to discuss their findings and refine the recommendations together with a broad range of stakeholders. The book they finally published was very well received.

*Situation D* comprises three projects (SWIP, DROUGHT-CH, IWAQA) that sought to provide decision-making tools based on better understanding of relevant socio-ecological systems. This required not only production of systems knowledge, but also transformation knowledge and—to a lesser extent—target knowledge. The relevant socio-ecological systems involved a high diversity of actors with different perspectives and needs. Some of the actors were interested in the projects, while others were relatively indifferent. Moreover, it was necessary to establish many new stakeholder contacts. For example, DROUGHT-CH aimed at developing an online platform to improve early warning of drought periods. The researchers mainly investigated soil humidity (systems knowledge). In addition, they conducted a needs analysis of a wide range of stakeholders and selected those most interested for further consultation. The results of consultation were used to establish a platform of value to different stakeholders. Only those stakeholders whose drought problems were related to soil humidity maintained their participation in the project.

In Situation D, a steadily rising degree of stakeholder interaction appeared necessary (similar to Situation C); however, higher overall degrees of stakeholder interaction were also required. As many different actors with different perspectives and needs were involved, and the intended contribution was a usable planning tool, it proved important to frame the project goals in consultation with concerned actors and to collaborate with these actors in the latter phases so as to co-produce implementable tools. In cases where relevant stakeholders were not engaged to sufficient degrees, the development and implementation of relevant tools proved difficult.

*Situation E* comprises three projects (WATER-CHANNELS, IWAGO, HYDROSERV) aimed at research capable of providing concrete recommendations for action. The projects intended to generate transformation knowledge similar to Situation D, but sought to go even further so as to reach action-relevant conclusions and/or stimulate societal debate. Moreover, compared with Situation D, the project topics in Situation E were more contested and some involved stakeholders who were relatively critical of the project goals.

For example, WATER-CHANNELS sought to identify the circumstances that make it is feasible to retain or reactivate traditional irrigation systems. They assumed that the existence of such irrigation systems is important for preservation of the cultural heritage of the region. However, the value of traditional irrigation systems and possible management options were contested among the stakeholders involved. Therefore, WATER-CHANNELS intended to generate new systems knowledge about ecological and socio-cultural impacts in order to stimulate societal debates about the maintenance and optimization of traditional irrigation systems. Although the issue is highly contested, the researchers did not intend to generate new target knowledge. Indeed, normative questions had already been focused on in earlier collaborations with the concerned actors. Instead, this project focused on production of transformation knowledge based on better systems understanding.

In such projects, it proved crucial to facilitate relatively high degrees of stakeholder involvement throughout all phases, including instances of knowledge co-production. As the projects sought to provide specific, actionable recommendations capable of implementation in contested situations, it was necessary to arrange intensive interactions with affected stakeholders and stakeholders with decision-making power. When stakeholders and their relations were not thoroughly analysed and/or stakeholders were not involved in the problem-framing phase, it proved difficult to generate knowledge that was relevant, acceptable, and implementable vis-à-vis concerned stakeholders.

*Situation F* comprises two projects (MONTANAQUA, AGWAM) that were very similar to Situation E projects, but emphasized production of target knowledge. For example, MONTANAQUA aimed at developing sustainable water governance strategies, in other words, concrete recommendations for action. With many different water users involved, the actor diversity could be considered high, and both the sustainability goals and the transformation strategies were highly contested among these actors. Therefore, MONTANAQUA viewed the joint development of future visions of sustainable water use (target knowledge) together with various stakeholders as a precondition for formulation of management strategies. The researchers already had some existing contacts, but many new collaborations had to be established and several stakeholder groups were not accustomed to cooperating with one another. General interest in the topic was relatively high, although some actors were indifferent because they had not yet experienced water-related problems, and others were critical because they feared that the new knowledge would undermine their privileged situation.

Both projects concluded that to fruitfully address the contested situations and to generate knowledge considered acceptable by different stakeholders, a high degree of stakeholder interaction would be needed allowing time for critical discourse on sustainability targets and underlying values. While *Situation C*–*E* projects generally require particularly high degrees of stakeholder interaction in the third phase so as to bring results to fruition, Situation F projects tend to require very intense stakeholder processes in the second phase in order to co-produce target knowledge.

## Discussion and conclusions

In this paper, we explored the stakeholder interaction processes of different research projects in order to identify what degrees of stakeholder interaction appeared most promising in particular situations. The research showed that very different degrees of stakeholder interaction appeared promising in different projects. But reflecting with the project representatives about the suitability of their approaches, we were able to identify six different degrees and modes of stakeholder interaction that appear promising in distinct situations. The situations are characterized by different manifestations of six criteria: (1) intended sustainability contribution, (2) knowledge forms to be produced, (3) contestation, (4) actor diversity, (5) actor interest, and (6) existing collaborations. Some situations called for consistently lower or higher intensity of interaction, others called for varying intensity according to different project phases, with higher levels required at the beginning, middle, and/or end. For example, projects seeking to induce actions for sustainability by generating novel and shared target knowledge on highly contested issues needed consistently higher degrees of stakeholder interaction (co-production) than projects aimed at generating systems knowledge related to rather uncontested issues (informing with feedback option). Furthermore, in situations where stakeholder collaborations were already well established and stakeholders had a high interest in obtaining scientific knowledge about an issue, lower degrees of interaction were fruitful (informing with feedback option), whereas in situations where researchers wanted to raise awareness about possible future risks that stakeholders had not previously been aware of, degrees of interactions needed to gradually increase (from informing to consultation).

We believe that systematic reflection on the insights of the present research can assist project designers in tailoring stakeholder interaction processes according to specific project goals and context conditions. Further, we believe the results can assist research funders in evaluating proposals. Indeed, discussing these insights with representatives of the research projects and funding bodies showed that three groups of actors found them especially useful:

- Researchers and coordinators of research programmes with limited prior experience in integrating stakeholders into research: These actors appreciated receiving guidance in systematically reflecting on their epistemological assumptions, impact goals, and context situations, so as to identify possible degrees of stakeholder interaction when developing research designs.
- Researchers or lecturers involved in education of graduate students: These actors appreciated having a structured way to explain different stakeholder interaction options to their students.
- Representatives of research funding bodies who evaluate research project proposals: These actors appreciated receiving guidance on how to evaluate a variety of different designs of stakeholder interaction.

However, the design of promising stakeholder interactions is a creative process—a process that cannot be fully grasped on the basis of standardized metrics such as those presented in this article. As shown above, application of the six criteria to specific situations does not work like a simple recipe book, since the criteria display various interrelations and researchers may reasonably decide to respond in one way or another. For example, different researchers may respond to similar context conditions with different sustainability-contribution goals: in situations where sustainability issues are highly contested and target knowledge is lacking, some researchers may seek to address the gap in target knowledge, while other researchers may opt to focus instead on relatively uncontested issues. Either option necessitates a different degree of stakeholder interaction. In this way, the criteria and situations identified above should be viewed as a resource for critical reflection and thinking, not as a precise decision tree.

Finally, the present research has certain limitations that should be addressed in order to further develop the insights into an assessment tool that can be readily applied by funding bodies:

- Evaluation time: our study took place towards the end of the research projects’ funding period and considered stakeholder processes and impacts that occurred during the research. However, sustainability impacts often require time to unfold.
- Scientist-centred valuations: our study only considered the perspectives of the scientists involved in the research projects, i.e. how they alone assessed the stakeholder interaction processes. However, when evaluating stakeholder-interaction processes, it is equally important to know the perspective of the societal stakeholders, of course, and how they perceive collaboration.
- Sample: our research was based on 16 research projects in the field of sustainable water management in Switzerland. While this sample is already relatively large for such qualitative studies, investigation of additional research projects in different contexts outside of Europe could reveal other manifestations and combinations.

In short, we consider reflection on the six criteria and their manifestations to be a valuable starting point for researchers and funders to find promising stakeholder interaction designs that deal responsibly with researchers’ and stakeholders’ limited time and financial resources. For researchers, reflection on the criteria is particularly helpful when framing new research projects aiming to contribute to sustainability transformations; and for research funding bodies, when evaluating such proposals. Formal application of the proposed approach in evaluating project proposals, however, would first require further testing of the approach.
